# Reliability of videotaped observational gait analysis in patients with orthopedic impairments

**DOI:** 10.1186/1471-2474-6-17

**Published:** 2005-03-17

**Authors:** Jaap J Brunnekreef, Caro JT van Uden, Steven van Moorsel, Jan GM Kooloos

**Affiliations:** 1 Department of Physical Therapy, Radboud University Nijmegen, P.O. Box 9101, 6500 HB Nijmegen, the Netherlands; 2 Department of Integrated Care, Research Institute Caphri, University Hospital Maastricht, P.O. Box 5800, 6202 AZ Maastricht, the Netherlands; 3 Department of General Practice, Research Institute Caphri, Maastricht University, P.O. Box 616, 6200 MD Maastricht, the Netherlands; 4 Department of Anatomy and Embryology, Radboud University Nijmegen, P.O. Box 9101, 6500 HB Nijmegen, the Netherlands

## Abstract

**Background:**

In clinical practice, visual gait observation is often used to determine gait disorders and to evaluate treatment. Several reliability studies on observational gait analysis have been described in the literature and generally showed moderate reliability. However, patients with orthopedic disorders have received little attention. The objective of this study is to determine the reliability levels of visual observation of gait in patients with orthopedic disorders.

**Methods:**

The gait of thirty patients referred to a physical therapist for gait treatment was videotaped. Ten raters, 4 experienced, 4 inexperienced and 2 experts, individually evaluated these videotaped gait patterns of the patients twice, by using a structured gait analysis form. Reliability levels were established by calculating the Intraclass Correlation Coefficient (ICC), using a two-way random design and based on absolute agreement.

**Results:**

The inter-rater reliability among experienced raters (ICC = 0.42; 95%CI: 0.38–0.46) was comparable to that of the inexperienced raters (ICC = 0.40; 95%CI: 0.36–0.44). The expert raters reached a higher inter-rater reliability level (ICC = 0.54; 95%CI: 0.48–0.60). The average intra-rater reliability of the experienced raters was 0.63 (ICCs ranging from 0.57 to 0.70). The inexperienced raters reached an average intra-rater reliability of 0.57 (ICCs ranging from 0.52 to 0.62). The two expert raters attained ICC values of 0.70 and 0.74 respectively.

**Conclusion:**

Structured visual gait observation by use of a gait analysis form as described in this study was found to be moderately reliable. Clinical experience appears to increase the reliability of visual gait analysis.

## Background

Patients exhibiting gait deviations caused by orthopedic impairments are often referred to a physical therapist for treatment. In order to determine treatment goals or to evaluate the effect of a therapeutic intervention, physical therapists visually observe the patient's gait [1-3]. This type of gait assessment is cost efficient, quick, and easy to use in comparison to computer-assisted gait analysis [1,3,4].

Several reliability studies on observational gait analysis have been described in the literature. These studies included patients with hemiplegia [5-7], amputation [8], neurological diseases [9], cerebral palsy [10], rheumatoid arthritis [11] and spinal cord injuries [12]. The outcomes of these studies are diverse. The inter-rater reliability score for 'live' observational gait analysis (OGA), varies from reasonable [9] to moderate – good [12]. The inter-rater reliability scores for videotaped observational gait analysis (VOGA) varies from moderate [11,13] to moderate – good [12], while others show that the intra-rater reliability of VOGA is poor [10], moderate [13] or good [12]. The results of the validity of 'live' and videotaped observation varies from reasonably good [5], to not valid [8] as well as valid and accurate [9,12]. Two other studies used VOGA, in which raters had the opportunity to look at a video in slow motion or freeze-frame. One of these studies, by Eastlack *et al*. [11], found only slight to moderate inter-rater reliability levels. The other study, by Hughes *et al*. [7], showed that only some parts of a hemiplegic gait analysis form show sufficient inter- and intra-rater reliability levels. All the above mentioned differences stem from a large variety in design, (amount and type of patients and raters, types of gait analysis forms, rating scales and types of statistical methods). Despite the numerous studies on observational gait analysis, patients with orthopedic impairments have received little attention.

In the Netherlands a gait analysis form has been developed which focuses mainly on orthopedic disorders [14]. Visual gait analysis with use of this gait analysis form is used by many physical therapists who practice gait training in patients with lower extremity orthopedic disorders. In addition, the use of this form is recommended by the Royal Dutch College of Physical Therapy for patients with chronic ankle sprain [14]. It is questionable, however, whether results from above described research concerning the reliability and validity of visual gait analysis in patients with neurological or other conditions can be extrapolated to patients with orthopedic problems. For example, gait deviations in patients with orthopedic impairments may result in less obvious gait deviations compared to patients with neurological disorders and may therefore be harder to identify visually.

The purpose of this present study is to determine the inter- and intra-rater reliability of videotaped observational gait analysis with use of an orthopedic gait analysis form when applied to a cohort of patients suffering from orthopedic impairments. In addition, this study determines how well the raters perform observational gait analysis by comparing their assessments with a criterion, based on the experts' opinion. In order to gain insight into how the results may give guidance to physical therapy treatment, this study also investigates which items on the gait analysis form, that have been considered to be disturbed by visual observation, receive high priority in the physical therapy treatment program according to the physical therapist who performs the visual gait analysis.

## Methods

### Patients

Thirty videotapes of patients' gait were selected from the archives of the department of Physical Therapy of the University Medical Center Nijmegen, the Netherlands. These videotapes involved patients who had been referred to a physical therapist for gait treatment. It is common practice at this department that prior to gait training therapy the gait of each patient is videotaped according to a standardized protocol.

The criteria for inclusion of the videotapes were: (1) the presence of mild to severe gait deviations due to an orthopedic impairment; (2) patient was wearing shorts or underwear to allow for a more accurate observation of the joint movement; (3) ability of a patient to walk 15 meters at least four times, twice in a semi-circle and twice in a straight line on a gymnasium floor; (4) and patient's written informed consent. The first thirty patients who complied with these criteria were included.

The group consisted of 15 male and 15 female patients with a mean age of 37.8 years (range: 15 to 62 years). The type of orthopedic impairments varied from status post hip, knee, ankle surgery (n = 8), status post hip or knee prosthesis (n = 6), status post femur, tibia or ankle fracture (n = 3) and traumatic or non-traumatic non-specific hip, knee or ankle pain (n = 13) (see Table 1).

**Table 1:** Patient characteristics (N = 30)

				Affected side	
				
Subject	Age (years)	Gender	Type of orthopedic impairment	Left side	Right side
1	25	F	Status post hip surgery		Hip
2	56	F	Status post hip prosthesis		Hip
3	48	M	Status post knee surgery	Knee	
4	19	F	Trauma induced non-specific knee pain		Knee
5	41	M	Status post hip surgery and prosthesis		Hip
6	62	F	Status post hip prosthesis	Hip	
7	37	M	Status post femur fracture		Hip/ Knee
8	49	M	Status post hip surgery and prosthesis	Hip	
9	15	F	Non-specific knee pain		Knee
10	33	M	Status post hip and knee prosthesis	Hip	Hip/ Knee
11	21	M	Status post ankle fracture	Ankle	Ankle
12	37	M	Status post ankle surgery	Ankle	
13	34	F	Non-specific knee pain		Knee
14	32	F	Status post knee surgery		Knee
15	19	F	Non-specific ankle pain	Ankle	Ankle
16	46	M	Non-specific knee pain		Knee
17	51	F	Non-specific knee pain		Knee
18	62	F	Status post hip surgery	Hip	
19	44	M	Status post hip surgery		Hip
20	21	M	Status post tibia fracture		Knee
21	33	F	Non-specific knee pain	Knee	
22	28	F	Status post hip surgery	Hip	
23	31	M	Status post ankle surgery		Ankle
24	60	F	Trauma induced non-specific knee pain	Knee	
25	52	M	Trauma induced non-specific knee pain	Knee	
26	50	F	Status post hip surgery and prosthesis	Hip	
27	21	F	Trauma induced non-specific ankle pain	Ankle	
28	41	M	Non- specific knee pain	Knee	Knee
29	49	M	Non- specific knee pain		Knee
30	16	M	Trauma induced non-specific knee pain		Knee

### Raters

Ten raters participated in this study, 4 inexperienced, 4 experienced and 2 experts. The inexperienced raters were two physical therapy students and two human movement science students. These inexperienced raters had no clinical experience in the analysis of gait deviations in orthopedic patients and never analyzed gait deviations by means of an observational gait analysis form.

The group of experienced raters consisted of four senior physical therapists who had all taken part and successfully completed a gait training course. All experienced raters had worked more than ten years as a physical therapist and had at least five years of experience in treating and analyzing gait deviations by means of an observational gait analysis form.

The two expert raters were two senior physical therapists, who were selected based on their exceptional skills and knowledge in the observation of gait deviations due to orthopedic impairments. They have considerable experience with treating patients with orthopedic gait disorders. In addition, these two physical therapists cooperatively developed the orthopedic gait analysis form used in this study and are instructors in a course in which participants are taught to treat and observe orthopedic gait deviations with a functional approach. All four experienced raters had taken part in this course.

### Design of the gait analysis form

The 12 items contained in the gait analysis form used in this study describe the trunk, arm, pelvis, hip, knee and ankle during the gait cycle (Table 2). In daily practice, the results of the visual gait analysis are used as a guide for treatment or to evaluate the effect of a therapeutic intervention.

**Table 2:** Orthopedic gait analysis form

				**STANCE PHASE**			**SWING PHASE**	
	**Item**	**Question**		**Early**	**Mid**	**Late**	**Early**	**Late**

**General**	**1**	Is a shortened stance phase present?	Left		Yes / No		NA	

			Right		Yes / No		NA	

**Trunk**	**2**	Is the trunk anterior to the hips?				Yes / No		
	**3**	Is the trunk posterior to the hips?				Yes / No		
	**4**	Is lateral flexion present?	Left		Yes / No		NA	
			Right		Yes / No		NA	
	**5**	Is arm-swing reduced?	Left			Yes / No		
			Right			Yes / No		

**Pelvis**	**6**	Is the posterior rotation excessive?	Left	NA		Yes / No	NA	
			Right	NA		Yes / No	NA	

**Hip**	**7**	Is the extension reduced?	Left	NA		Yes / No	NA	
			Right	NA		Yes / No	NA	

**Knee**	**8**	Is the extension reduced?	Left	NA			NA	Yes / No
			Right	NA			NA	Yes / No
	**9**	Is the flexion movement absent ?	Left	Yes / No	NA		NA	
			Right	Yes / No	NA		NA	
	**10**	Is the flexion reduced?	Left	Yes / No	NA		NA	
			Right	Yes / No	NA		NA	
	**11**	Is the extension absent?	Left	NA	Yes / No	NA	NA	
			Right	NA		Yes / No	NA	

**Ankle**	**12**	Is the plantar flexion reduced?	Left	NA		Yes / No	NA	
			Right	NA		Yes / No	NA	

NA = not applicable

### Visual gait analysis

The gait pattern was analyzed from a lateral (both sides), anterior and posterior view at each of the three sub-phases of stance and the two sub-phases of swing. *Early stance *was defined as the combined phases of initial contact and loading response. In this phase, the ankle moves from heel contact to foot contact, while the knee is flexed to absorb the shock of limb loading. *Mid-stance *was defined as the phase of foot contact to heel rise, during this phase the trunk progresses over a single stable limb. *Late stance *was defined as the combined phases of terminal stance and pre-swing, in which heel-rise and toe-off occurs. *Early swing *was defined as toe-off until to the swing leg reaches the stationary leg. *Late swing *was defined as the combined phases of mid swing and terminal swing. In this phase, the moving leg passes the stationary leg and the knee extends as the limb prepares to take the load at initial contact.

### Videotape recording

All patients were recorded from a lateral view (both sides) while walking 15 meters in a semi-circle (radius approximately 10 m) at a comfortable self-selected walking speed. We used a semi-circle in order to be able to observe the patient's gait in the sagittal plane from one position. The anterior and posterior views were videotaped while the patient walked five meters toward and away from the camera.

The collected videos were edited with use of the computer program adobe premiere 6.0 (Adobe systems^®^). Manufactured videos were reduced into a one-minute film-clip in which the patient's gait could be viewed in the lateral and frontal plane. Subsequently, these videos were converted to analog format again, so that they could be played by a regular video player. Sampling frequency was 24 Hz.

### Rater instructions

To ensure visual assessment of gait based on comparable criteria, all raters received standardized information about normal gait kinematics prior to the rating sessions (Table 3). Raters were required to use this information during the rating sessions. Before each session, raters viewed a videotaped gait sequence of a non-participating patient and a healthy subject. All raters started rating after they felt completely comfortable with rating the videos.

**Table 3:** Normal joint-angles during stance and swing phase. Range of motion summary in the sagittal plane measured in degrees.

**Phase of the gait cycle**	**STANCE PHASE**			**SWING PHASE**	
	
	**Early **0 – 10% GC.	**Mid **10 – 30% GC.	**Late **30 – 60% GC.	**Early **60 – 70% GC.	**Late **70 – 100% GC.
**Trunk**	Positioned above the hip			Positioned above the hip	

**Pelvis**	5° forward rotation	0°	5° backward rotation	5° backward rotation	5° forward rotation
**Hip**	25° flexion	0°	30–50% GC: 10° extension 50–60% GC: 0°	15° flexion	25° flexion
**Knee**	20° flexion	0°	40° flexion	60° flexion	0°
**Ankle**	10° PF	10° DF	20° PF	10° PF	0°

GC = Gait Cycle, PF = Plantar Flexion, DF = Dorsal Flexion [1]

### Rating procedure

The rating session took place in an isolated room in which each rater individually assessed the videotaped gait-patterns of 30 patients twice, with a minimum interval between the two rating sessions of 3 weeks, in order to reduce the effect of recognition. Raters had to rate each item of the form as present or absent. Both legs were assessed and were dealt with in the statistical analysis as independent ratings. Each rater was permitted to view the videotape in slow motion or freeze-frame, allowing the raters to more closely inspect the patient's gait. Each rater was able to rate the patient's gait as many times as necessary until they were satisfied with their rating. The rating of the 30 patients was spread out over two days and a single session lasted for a maximum of two hours. All videos were put in a randomized order to prevent the raters from recognizing the patient and recalling their scores from the last session. The randomization was done through the use of dice and was concealed from all raters.

Raters were also asked to assign priority levels (high or low priority) to the items they scored as disturbed, with respect to a physical therapy treatment program. In other words, which items would receive important attention in the physical therapy intervention if the rater was going to treat this patient for his or her gait disorder.

### Level of performance

In order to determine the level of performance of observational gait analysis of all experienced and inexperienced raters, we compared their ratings with a criterion. This gives us an indication about how well the raters were capable in performing visual gait analysis. The criterion was attained during a consensus session of the two expert raters: After individually assessing the 30 patients for the second time, the two expert raters jointly observed the videotaped gait of all 30 patients for the third time.

### Data analysis

Inter- and intra-rater reliability levels were assessed by using Intraclass Correlation Coefficients (ICCs), validated for use with multiple raters and calculated in a two-way random model based on absolute agreement. We used ICCs because it has been shown that with data that are rated as a dichotomy, the ICC is equivalent to measures of nominal agreement, simplifying computation in cases where more than two raters are involved [15]. In addition, the ICC computation also provides us with an estimate of accuracy (95% CI) of the reliability levels. The level of performance (quality of assessment) was obtained by comparing the joint assessment of the expert raters to each individual, also using reliability analyses with use of ICCs. Agreement strengths for ICC values have been classified as follows: <0 = poor; 0 – 0.20 = slight, 0.21 – 0.40 = fair; 0.41 – 0.60 = moderate; 0.61 – 0.80 = substantial and 0.81 – 1.00 = almost perfect [16]. All analyses were performed with use of SPSS 11.0.1.

## Results

### Inter-rater reliability

The inter-rater reliability among experienced raters was 0.42 (95%CI: 0.38–0.46). This level of reliability is comparable to the inter-rater reliability of in-experienced raters, which reached an ICC value of 0.40 (95%CI: 0.36–0.44). The expert raters reached the highest inter-rater reliability (ICC: 0.54 (95%CI: 0.48–0.60)).

There were no differences in inter-rater reliability between the first and second rating session of all three groups separately, based on the overlap of 95% confidence intervals.

### Intra-rater reliability

The average intra-rater reliability of the experienced raters was 0.63 (ranging from 0.57 to 0.70). The inexperienced raters reached an average intra-rater reliability of 0.57(ranging from 0.52 to 0.62). The two expert raters attained ICC values of 0.70 and 0.74 respectively.

### Level of performance

The agreement between the outcome of the joint assessment of the expert raters (criterion) and those of the individual experienced raters ranged from 0.43 to 0.55 with an average ICC value of 0.48. The inexperienced raterrs attained agreement levels ranging from 0.41 to 0.55, with an average of 0.49. There is no difference in the level of performance of visual gait assessments of experienced or inexperienced raters, when compared to the experts' opinion.

### Reliability levels for each item separately

The inter-rater reliability per item on the gait analysis form between the two experts is generally moderate to substantial (see Table 4). However, two items in particular, showed low agreement levels. These are flexion of the knee during early stance (item 9) and posture of the trunk during walking (item 2) (for both: ICC = 0.33). With respect to the experienced and inexperienced raters, the visual observation of the lateral flexion of the trunk (item 4), the arm swing (item 5) and the knee extension in the late swing phase (item 8) showed the highest inter-rater reliability levels (all ICC-values > 0.50).

**Table 4:** Reliability of the gait analysis list per item

		**Inter-rater reliability^1^**			**Intra-rater reliability^2^**		
	**Item**	**Expert (n = 2)**	**Experienced (n = 4)**	**Inexperienced (n = 4)**	**Expert (n = 2)**	**Experienced (n = 4)**	**Inexperienced (n = 4)**

		ICC (95% CI)	ICC (95% CI)	ICC (95% CI)	Mean ICC (range)	Mean ICC (range)	Mean ICC (range)
**General**	**1**	0.62 (0.43 – 0.76)	0.25 (0.12 – 0.39)	0.26 (0.14 – 0.40)	0.86 (0.82 – 0.89)	0.54 (0.32 – 0.83)	0.50 (0.36 – 0.65)
**Trunk**	**2**	0.33 (0.01 – 0.61)	0.25 (0.12 – 0.39)	0.41 (0.22 – 0.61)	0.87 (0.74 – 1.00)	0.81 (0.64 – 1.00)	0.53 (0.37 – 0.64)
	**3**	-	-	-	-	-	-
	**4**	0.66 (0.50 – 0.78)	0.58 (0.46 – 0.70)	0.52 (0.39 – 0.65)	0.82 (0.69 – 0.95)	0.68 (0.49 – 0.86)	0.74 (0.66 – 0.84)
	**5**	0.48 (0.17 – 0.68)	0.53 (0.40 – 0.66)	0.55 (0.40 – 0.69)	0.75 (0.70 – 0.79)	0.75 (0.61 – 0.82)	0.81 (0.74 – 0.88)

**Pelvis**	**6**	0.58 (0.38 – 0.73)	0.19 (0.08 – 0.34)	0.33 (0.20 – 0.47)	0.65 (0.53 – 0.76)	0.13 (-0.07 – 0.61)	0.45 (0.27 – 0.61)

**Hip**	**7**	0.52 (0.23 – 0.71)	0.43 (0.28 – 0.57)	0.24 (0.11 – 0.39)	0.63 (0.59 – 0.67)	0.59 (0.35 – 0.72)	0.47 (0.14 – 0.67)

**Knee**	**8**	0.58 (0.34 – 0.73)	0.58 (0.45 – 0.70)	0.60 (0.48 – 0.71)	0.66 (0.62 – 0.69)	0.65 (0.49 – 0.82)	0.63 (0.48 – 0.76)
	**9**	0.33 (0.07 – 0.54)	0.45 (0.32 – 0.59)	0.16 (0.05 – 0.30)	0.82 (0.76 – 0.88)	0.58 (0.44 – 0.72)	0.36 (0.10 – 0.55)
	**10**	0.51 (0.30 – 0.68)	0.23 (0.10 – 0.38)	0.41 (0.28 – 0.55)	0.82 (0.70 – 0.94)	0.42 (0.02 – 0.65)	0.54 (0.50 – 0.64)
	**11**	0.40 (0.16 – 0.59)	0.29 (0.15 – 0.44)	0.36 (0.23 – 0.50)	0.52 (0.42 – 0.61)	0.58 (0.47 – 0.63)	0.22 (0.00 – 0.54)

**Ankle**	**12**	0.52 (0.27 – 0.70)	0.30 (0.17 – 0.45)	0.20 (0.09 – 0.35)	0.66 (0.62 – 0.70)	0.30 (0.16 – 0.46)	0.37 (0.17 – 0.67)

The intra-rater reliability levels with respect to the visual gait assessments by expert raters were generally higher compared to the experienced and inexperienced raters. With regard to five items intra-rater reliability was good (>0.80). Only one item, extension movement of the knee during mid stance, had an ICC value for intra-rater reliability of less than 0.6. The experienced raters were able to attain good intra-rater reliability for item 2, posture of the trunk during walking (ICC = 0.81). Three items reached substantial intra-rater reliability (item 4, 5, and 8). Two items of the gait analysis form, pelvis rotation and ankle movement during late stance, were not intra-rater reliable (ICC < 0.40). The inexperienced raters reached the highest intra-rater reliability for the assessment of arm swing during walking (ICC = 0.81). Three items had inadequate intra-rater reliability levels; flexion of the knee in early stance (ICC = 0.36), extension of the knee in mid stance (ICC = 0.22), and ankle movement during the late stance phase (ICC = 0.37).

No reliability score was obtained from item 3, which describes a trunk position behind the hips, because this item was observed only once.

### Priority level with respect to physical therapy treatment

On average, with respect to all items, in about a quarter of the cases items were judged to be disturbed by the expert and experienced raters (see Table 5). Except for item three which was considered disturbed only once in the group of experienced raters. Both expert and experienced raters would give hip, knee and ankle movements, which were judged as being disturbed, generally high priority if they were to treat the patient. Expert raters also gave a shortened stance phase of either one of the legs, and an excessive lateral flexion of the trunk high priority, in contrast to the experienced raters for whom these items received generally a low priority. The other items such as movement of the pelvis, arm swing, and position of the trunk (flexed or extended) received generally low priorities in a potential physical therapy intervention.

**Table 5:** Treatment priority per item when scored as disturbed.

		**Expert raters**			**Experienced raters**		
			**Treatment priority^b^**			**Treatment priority^b^**	

	Item	*Times scored as disturbed*^a^	**High**	**Low**	*Times scored as disturbed^a^*	**High**	**Low**

**General**	**1**	*15,0%*	72,2%	27,8%	*13,8%*	21,2%	78,8%

**Trunk**	**2**	*16,7%*	20,0%	80,0%	*15,8%*	47,4%	52,6%
	**3**	*0,0%*	-	-	*0,8%*	100,0%	0,0%
	**4**	*26,7%*	71,9%	28,1%	*26,3%*	54,0%	46,0%
	**5**	*50,8%*	55,7%	44,3%	*40,4%*	24,7%	75,3%

**Pelvis**	**6**	*22,5%*	44,4%	55,6%	*7,9%*	26,3%	73,7%

**Hip**	**7**	*42,5%*	82,4%	17,6%	*26,7%*	82,8%	17,2%

**Knee**	**8**	*26,7%*	59,4%	40,6%	*25,4%*	75,4%	24,6%
	**9**	*20,0%*	95,8%	4,2%	*41,7%*	98,0%	2,0%
	**10**	*24,2%*	100,0%	0,0%	*52,9%*	99,2%	0,8%
	**11**	*45,8%*	67,3%	32,7%	*26,3%*	92,1%	7,9%

**Ankle**	**12**	*54,2%*	83,1%	16,9%	*35,8%*	96,5%	3,5%

^a ^This number indicates how many times raters scored this item as being disturbed.

^b ^When raters scored an item as being disturbed they were asked to indicate whether this item would receive high or low priority in their physical therapy treatment program with respect to the patients gait disorder.

## Discussion

The results of this study indicate a moderate reliability of observational gait analysis in patients with orthopedic gait disorders while using a structured gait analysis form. In addition, the observation of only three items of the gait analysis form reached substantial levels of inter-rater reliability. These were related to lateral movements of the trunk, arm swing, and the movement of the knee just before heel strike.

This study shows comparable results with similar studies on observational gait analysis in different patient categories. Studies on visual gait analysis that show high reliability levels, generally focused on patients exhibiting severe neurological pathology. Severe neurological pathology causes grossly larger gait deviations, which makes potential gait deviations easier to recognize. Furthermore, most of the gait analysis forms being used contain easy observable items. With respect to the present study, the highest agreement levels are reached on items that are considered easy observable: the lateral flexion of the trunk, the arm swing and the knee extension in the late swing phase. Items that are considered more difficult to observe, like the pelvis rotation and the plantar flexion of the ankle in the late stance phase, scored lower agreement levels. Minute gait deviations displayed by the patients in this study lead to difficult observable items, explaining the moderate reliability level found in this present study.

Another explanation for the moderate results may be that some of the patients in this study displayed an inconsistent gait pattern. This means that, despite the accuracy with which the videos were collected in this study, still some participants performed a slight variability in their gait pattern. This results in small gait deviations present during a few steps and absent a couple of steps later, so when raters do not observe the same gait cycles, differences occur. This might explain relatively low inter- and intra-rater reliability levels, even when raters were 'right' in their assessment. To correct for this disturbance we believe that a gait deviation should only be defined as abnormal when the patient repeats the deviation in a series of gait cycles. This will increase reliability levels of the videotaped observational gait analysis. On the other hand, inconsistent gait patterns are of minor importance during 'live' observation or videotaped gait observation without the opportunity for freeze-frame or slow-motion. In that case more gait cycles are observed, leading to a situation in which an average of the inconsistencies is scored. This consideration is supported by the fact the reliability of gait analysis without the opportunity for freeze-frame or slow-motion is not always found to be worse [12,13].

A weakness of this study is that we have not included an objective standard to assess the validity of raters' visual observations. Nevertheless, we tried to gain insight in raters' performance by using a criterion, which was accomplished during a joint rating session by the two expert physical therapists.

According to this study, experience in gait observation does not improve the reliability of this observation. Inexperienced raters achieve a comparable reliability level to experienced raters. However, expert raters accomplish significant better reliability levels of visual gait observation compared to experienced and inexperienced raters. In other words, some experience does not improve observation skills, but a lot more does.

We have shown that not all movements of body segments during gait can be observed with similar reliability levels. The visual observation of only three items proved to be substantially reliable. This indicates that one should bear in mind when using this 12-item gait analysis form that nine of these items are at the best moderately reliable. However, the results of this study indicate that for at least four items the intra-rater reliability levels are substantial to good (items 2, 4, 5 and 8). Expert raters showed the least variability between the first and second session; five items showed to have a mean intra-rater reliability level that is considered good (ICC > 0.80).

The results of this study suggest that a brief introduction in normal gait kinematics in inexperienced raters gives comparable reliability levels of observational gait analysis in patients with orthopedic impairments compared to experienced physical therapists, who have worked for several years with patients with gait disorders. However, expert raters – those that work significantly more intensive with patients with gait disorders – accomplish higher reliability levels.

As mentioned in the methods section, the gait analysis form used in this study is also used in daily practice to guide the treatment of the patient's gait disorder. In the physical therapist's treatment program, some items on the form will obviously receive higher priority than others. The results of this study show that physical therapists mainly focus their intervention on movement disorders of the lower extremity. However, the expert raters also report to give priority to asymmetry of the stance phase and excessive lateral flexion of the trunk during gait. Of the three items in this study that achieved the highest reliability levels, only the movement of the knee received generally a high priority in the treatment program of experienced raters. This implies that experienced raters will mainly focus their treatment on items that have generally a low inter- and intrarater reliability.

## Conclusion

Structured visual observation of a patient's gait by use of a gait analysis form as described in this study is found to be only moderately reliable, but may be a useful guide to the physical therapist in setting up a gait training or exercise therapy program. Intra-rater levels have shown that visual gait analysis will supply the observer with a fair indication of changes in a person's gait. However, to evaluate the effect of an intervention on a patient's gait we recommend more objective instrumentation which has been proven reliable and valid.

## Competing interests

The author(s) declare that they have no competing interests.

## Authors' contributions

JB carried out the data analysis, participated in the design of the study and drafted the manuscript. CvU participated in the design and coordination of the study, assisted with statistical analysis, and helped to draft the manuscript. SvM participated in the design of the study and supplied the videotapes. JK participated in the design and coordination of the study. All authors read and approved the final manuscript.

## Pre-publication history

The pre-publication history for this paper can be accessed here:



## References

[B1] Coutts F (1999). Gait analysis in the therapeutic environment. Man Ther.

[B2] Kopf A, Pawelka S, Kranzl A (1998). Clinical gait analysis--methods, limitations and possible applications. Acta Med Austriaca.

[B3] Malouin F, Craik RL and Oatis CA (1995). Observational gait analysis: normal and pathological function. Gait Analysis, Theory and Application.

[B4] Harris GF, Wertsch JJ (1994). Procedures for gait analysis. Arch Phys Med Rehabil.

[B5] Miyazaki S, Kubota T (1984). Quantification of gait abnormalities on the basis of continuous foot-force measurement: correlation between quantitative indices and visual rating. Med Biol Eng Comput.

[B6] Goodkin R, Diller L (1973). Reliability among physical therapists in diagnosis and treatment of gait deviations in hemiplegics. Percept Mot Skills.

[B7] Hughes KA, Bell F (1994). Visual assessment of hemiplegic gait following stroke: pilot study. Arch Phys Med Rehabil.

[B8] Saleh M, Murdoch G (1985). In defence of gait analysis. Observation and measurement in gait assessment. J Bone Joint Surg Br.

[B9] Lord SE, Halligan PW, Wade DT (1998). Visual gait analysis: the development of a clinical assessment and scale. Clin Rehabil.

[B10] de Bruin H, Russell DJ, Latter JE, Sadler JT (1982). Angle-angle diagrams in monitoring and quantification of gait patterns for children with cerebral palsy. Am J Phys Med.

[B11] Eastlack ME, Arvidson J, Snyder-Mackler L, Danoff JV, McGarvey CL (1991). Interrater reliability of videotaped observational gait-analysis assessments. Phys Ther.

[B12] Field-Fote EC, Fluet GG, Schafer SD, Schneider EM, Smith R, Downey PA, Ruhl CD (2001). The Spinal Cord Injury Functional Ambulation Inventory (SCI-FAI). J Rehabil Med.

[B13] Krebs DE, Edelstein JE, Fishman S (1985). Reliability of observational kinematic gait analysis. Phys Ther.

[B14] (2003). Royal Dutch College of Physical Therapy. Guideline for chronic ankle sprain.

[B15] Portney LG, Watkins WP (1993). Foundations of clinical research: applications to practice.

[B16] Landis JR, Koch GG (1977). The measurement of observer agreement for categorical data. Biometrics.

